# Alternative Splicing of TAF6: Downstream Transcriptome Impacts and Upstream RNA Splice Control Elements

**DOI:** 10.1371/journal.pone.0102399

**Published:** 2014-07-15

**Authors:** Catherine Kamtchueng, Marie-Éve Stébenne, Aurélie Delannoy, Emmanuelle Wilhelm, Hélène Léger, Arndt G. Benecke, Brendan Bell

**Affiliations:** 1 RNA Group, Département de microbiologie et d'infectiologie, Faculté de médecine et sciences de la santé, Université de Sherbrooke, and Centre de recherche du CHUS, Pavillon de recherche appliquée sur le cancer, 3201 rue Jean-Migneault, Sherbrooke, Québec, Canada; 2 Institut des Hautes Etudes Scientifiques, Centre National de la Recherche Scientifique, 35 route de Chartres, Bures sur Yvette, France; 3 Université Pierre et Marie Curie, UMR8246 CNRS, 7 quai Saint Bernard, Paris, France; International Centre for Genetic Engineering and Biotechnology, Italy

## Abstract

The TAF6δ pathway of apoptosis can dictate life versus death decisions independently of the status of p53 tumor suppressor. TAF6δ is an inducible pro-apoptotic subunit of the general RNA polymerase II (Pol II) transcription factor TFIID. Alternative splice site choice of TAF6δ has been shown to be a pivotal event in triggering death via the TAF6δ pathway, yet nothing is currently known about the mechanisms that promote TAF6δ splicing. Furthermore the transcriptome impact of the gain of function of TAF6δ versus the loss of function of the major TAF6α splice form remains undefined. Here we employ comparative microarray analysis to show that TAF6δ drives a transcriptome profile distinct from that resulting from depletion of TAF6α. To define the *cis*-acting RNA elements responsible for TAF6δ alternative splicing we performed a mutational analysis of a TAF6 minigene system. The data point to several new RNA elements that can modulate TAF6δ and also reveal a role for RNA secondary structure in the selection of TAF6δ.

## Introduction

The TAF6δ pathway of apoptosis (Fig. S1) can control cell death versus life decisions of human cells [1], [2], [3]. TAF6δ is a splice variant of the TAF6 protein that is a core subunit of the general RNA polymerase II (Pol II) transcription factor, TFIID [4], [5]. TFIID nucleates the formation of the Pol II pre-initiation complex and therefore represents a highly regulated step in the gene expression pathway of protein-coding genes [6]. TFIID is the major core promoter recognition complex of the Pol II machinery and consists of TATA-binding protein (TBP) and a constellation of approximately 14 TBP-associated factors (TAFs) [7]. TAF6δ is an inducible pro-apoptotic isoform of TAF6 that lacks 10 amino acids in its histone-fold domain. In contrast to the major TAF6α isoform, TAF6δ cannot interact with TAF9 and instead forms a TAF9-lacking complex termed TFIIDπ that drives a pro-apoptotic gene expression [2]. The TAF6δ pathway has emerged as a model system to investigate the mechanisms that transduce extracellular signals to trigger cellular suicide by impinging on the basal Pol II machinery. Moreover, because the TAF6δ pathway induces cell death independently of p53 [3], it represents a potential therapeutic target of strategic value for the killing of tumor cells that frequently lack functional p53 [8].

Modified antisense RNA oligonucleotides that force the splicing machinery to switch from producing a majority of TAF6α to producing a majority of TAF6δ in living cells trigger apoptosis demonstrating that changes in alternative splicing can trigger the TAF6δ pathway of programmed cell death [2], [3]. Alternative splicing plays a major role in proteomic diversification [9], [10]. In the case of programmed cell death, alternative splicing can control cell life versus death decisions by regulating the balance of anti-apoptotic versus pro-apoptotic splice variants of genes within cell death pathways [11]. *Cis*-acting RNA elements can either enhance or silence the selection of alternative splice sites by the spliceosome to control splice site decisions [12]. These elements are classified upon their effect on a given splicing event and their location. Regulatory *cis*-acting RNA elements thus include exonic splicing enhancers (ESE), exonic splicing silencers (ESS), intronic splicing enhancers (ISE), and intronic splicing silencers (ISS). These *cis*-acting RNA elements act to recruit *trans*-acting protein factors, often from the SR protein family [13] or the hnRNP family [14]. Layered upon the network of RNA-protein interactions that underpin alternative splicing decisions is the key role of RNA secondary structure within the pre-mRNA that has an impact on splice site recognition by the spliceosome as well as on RNA-protein interactions [15], [16], [17], [18].

One important challenge in the study of all alternative splice events is to define the relative biological impact of the gain of the alternative splice form versus the loss of the constitutive form. While the induction of alternative splice variants often have important biological effects, in extreme cases alternative splicing serves only to dampen gene expression, as is the case when these events are coupled to the nonsense-mediated decay pathway [19], [20]. To shed light on the mechanism controlling the TAF6δ pathway of apoptosis, here we have compared the transcriptome impacts of loss of function of the major TAF6α splice variant via siRNA depletion versus those resulting from the induction of the pro-apoptotic TAF6δ splice variant. The results reveal an essential function for TAF6δ induction in the reprogramming of a specific pro-apoptotic transcriptome landscape. Despite the importance of inducible TAF6δ expression, nothing is currently known about the mechanisms governing alternative TAF6δ splicing. We therefore developed and validated a minigene system for the mutational dissection of TAF6 *cis*-acting RNA elements. We report here the first identification of RNA elements that can influence splicing of TAF6δ.

## Materials and Methods

### Cell culture

Hela ws cell line was maintained in culture in Dulbecco's modified Eagle's medium supplemented with 2.5% fetal calf serum and 2.5% calf serum.

### Plasmids

To construct the TAF6 minigene (pTAF6mg), the genomic region of TAF6 containing exon 2 to exon 3 was amplified by PCR from HeLa cell genomic DNA with primers 5′-AAAAAGGGATCCCATGGGCATCGCCCAGATTCAGG-3′ (forward) and 5′-AAAAAGGAATTCCAAGGCGTAGTCAATGTCACTGG-3′ (reverse). The PCR product was ligated into pTZ57R/T (Fermentas). The new plasmid was digested with EcoRI and BamHI and the TAF6 fragment was inserted into the same sites of pcDNA3.1+. The mutated minigenes were created by PCR mutagenesis using Pfu DNA polymerase with specific primers bearing mutations [21] (all sequences of oligonucleotides used in this study are listed in Table S1).

### Transfections

Dicer substrate (dsi) RNA 5′-rGrGrArGrUrGrUrCrCrArGrArArGrUrArCrArUrCrGrUrGGT-3′ (T6-1) and 5′-rCrGrCrUrArArGrCrGrGrArArGrGrArArGrUrUrGrUrArGAT-3′ (T6-2) were employed to deplete all known splice variants of TAF6. dsiRNA were transfected at a final concentration of 10 nM with lipofectamine 2000 (Invitrogen) as a delivery agent (1.6 µl/ml) according to the manufacturer's recommendations. 250 to 300 ng of wild-type or mutant TAF6 minigene were transfected with 1 µl DMRIE-C (Invitrogen) per well in 24 well plate according to the manufacturer's recommendations. Cells were transfected by dsiRNA T6-1 two times. The second transfection was performed 24 h after the first one and culture was maintained for a total of 48 or 72 h before harvesting cells for RNA extraction (RNeasy Qiagen) for microarray analysis or 64 h for protein analysis. All transfections were performed with OptiMEM medium (Invitrogen). Each transfection experiment was repeated three times.

### Viability assay by methylene blue staining

HeLa cells were split in 24 well plates at a concentration of 75 000 cells/well and transfected 12 hours later with 10 nM dsiRNA combined with lipofectamine 2000 (Invitrogen) as recommended by the supplier. dsiRNA were transfected again 24 and 48 h after the first transfection. The culture was maintained for a total of 4 days after the first transfection. The culture medium was removed and the cell monolayer was washed carefully with 500 µl PBS. Cells were stained for 30 min at RT by the addition of 200 µl of a solution containing 5 mg/ml of methylene blue in 50% ethanol. The plate was carefully and extensively washed with water until no blue stain remained in the water. The plate was air dried completely. 500 µl of a PBS solution containing 10 mg/ml N-lauroyl sarcosine (Sigma L-5125) was added to each well. Lysis was performed for 1 h at RT. 100 µl of each lysate was used to measure absorbance at 595 nm (A515 nm = control), corresponding to methylene blue incorporation and cell content.

### RT-PCR

Total RNA was extracted from cells using Trizol (Invitrogen) according to the manufacturer's recommendations. RNA was treated with 1 unit of DNase I (Promega) for 30 minutes at 37°C to remove any contaminating DNA. 1 µg of total RNA was reverse transcribed using MMuLV reverse transcriptase. Specific oligonucleotides for endogenous TAF6 (forward 5′-ATGGGCATCGCCCAGATTCAGG-3′ and reverse 5′-AAGGCGTAGTCAATGTCACTGG-3′) and exogenous TAF6 minigene constructs (forward 5′-ATGGGCATCGCCCAGATTCAGG-3′ and reverse 5′-AATAGCGATCCACGCGACTAGTGG-3′) were used for PCR amplification of 1/10 of the total cDNA (25 cycles, 1 min at 94°C, 45 sec at 58°C, 50 sec at 68°C, initial step 3 min at 95°C, final extension 5 min at 68°C), using Taq DNA polymerase. The splicing isoforms were quantified by capillary electrophoresis on a BioAnalyser 2100 (Agilent) according to the manufacturer's instructions.

### Western blot analysis

After treatment as described, cells were lysed in 1.5× laemmli sample buffer, sonicated and electrophoresed on 7.5 and 12% SDS-PAGE before electro-transfer to PVDF membrane. Blots were probed with previously described antibodies against TAF6α, TAF6δ or TBP [1] followed by goat anti-mouse HRP-conjugate secondary antibodies (Jackson Immunoresearch Laboratories) before enhanced chemiluminescence detection. Mouse monoclonal antibodies that detect total TAF6 proteins levels were from BD transduction laboratories #610304.

### Microarrays

RNA isolation and quality control were performed as previously described [2], [22] from cells treated with siRNA T6-1. Applied Biosystems HGS V2 arrays [23] were hybridized, washed, and exposed according to the protocols of the technology provider. Raw data were quality controlled [24], and normalized using NeONORM (k-parameter = 0.2) [25]. Longitudinal analysis was performed using a Kohonen-maps based classifier as described in [26], using the CDS statistical test [27]. The data are freely accessible through the MACE database at http://mace.ihes.fr under accession no. (maceid): 2732656872.

## Results

### SiRNA-mediated depletion of TAF6 causes a loss of viability in human cells

**Figure 1:**
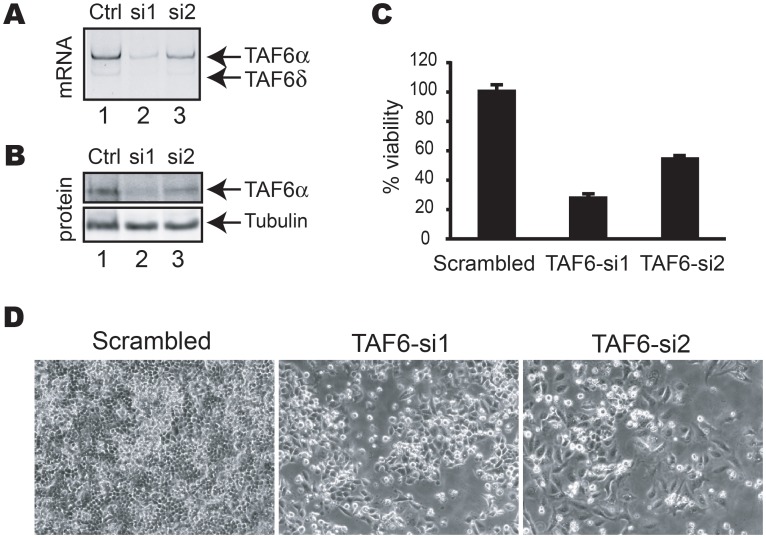
TAF6 is essential for human cell viability. (A) PCR analysis of TAF6 mRNA levels 72 hours post-transfection with siRNAs targeting all TAF6 mRNAs. (B) Total cell extracts from HeLa cells transfected with the indicated siRNAs for 72 hours were separated by SDS-PAGE subject to Western blot analysis with monoclonal antibodies specific for TAF6α and tubulin as a loading control. (C) The viability of HeLa cells transfected with siRNA directed against TAF6 was measured 4 days post-transfection by methylene blue staining (see materials and methods). Viability (y-axis) is expressed relative to that of cells transfected with control siRNA. (D) TAF6 depletion results in loss of HeLa cell viability. HeLa cells were treated for 4 days with control siRNA (panel 1), or siRNA that target the TAF6 mRNA (panels 2 & 3). Cells were photographed with a phase-contrast microscope.

We have previously analyzed the transcriptome impact of the induction of TAF6δ by using microarray experiments [2]. To define the impact of the loss of total TAF6 protein in human cells we performed transcriptome-wide analysis of gene expression following depletion of total TAF6 proteins by siRNA. Importantly, the minor TAF6δ protein isoform is not expressed at detectable levels in HeLa cells under normal growth conditions, so that the effects of the siRNA cannot be due to significant reductions in TAF6δ protein levels [2], [3] (see also Fig. S2). siRNAs were designed and validated for their ability to reduce TAF6 protein levels. Two siRNAs resulted in reduction of TAF6 mRNA levels (Fig. 1A) and protein levels (Fig. 1B). Quantification of TAF6α protein levels from three independent experiments using the ImageJ software package (http://rsbweb.nih.gov/ij/) indicated that si1 and si2 typically reduced TAF6α protein levels by 70–80% and 40–60%, respectively. To determine the impact of the loss of total TAF6 protein on cell viability we employed the methylene blue colorimetric assay (see materials and methods). Both siRNAs that depleted TAF6 resulted in a concomitant loss of cell viability (Fig. 1C–D). We conclude that TAF6 expression is essential for cell viability in human cells, extending previous results demonstrating that TAF6 is essential for viability in distinct organisms including *S. cerevisae* [28], [29], Drosophila [30] and zebrafish [31]. We next used si1, the siRNA that most efficiently reduced total TAF6 expression and resulted in the highest loss of viability, to further determine the impact of knock-down of TAF6 on the transcriptome using microarray analysis. Total RNA was isolated from HeLa cells treated with siRNA directed against TAF6 and from cells treated with control siRNA for microarray analysis [2], [22], [32]. Two time points (48 and 72 hours) were chosen for transcriptome analysis to ensure the detection of the broadest possible number of TAF6-dependent transcripts as well as to ensure measurements before the onset of massive cell death. The levels of TAF6 mRNA were internally controlled in the transcriptome analysis by probes for *taf6* and showed at least 80% reduction at both 48 and 72 hours post-transfection (data not shown).

### Distinct transcriptome impacts of depleting TAF6α versus inducing TAF6δ

**Figure 2:**
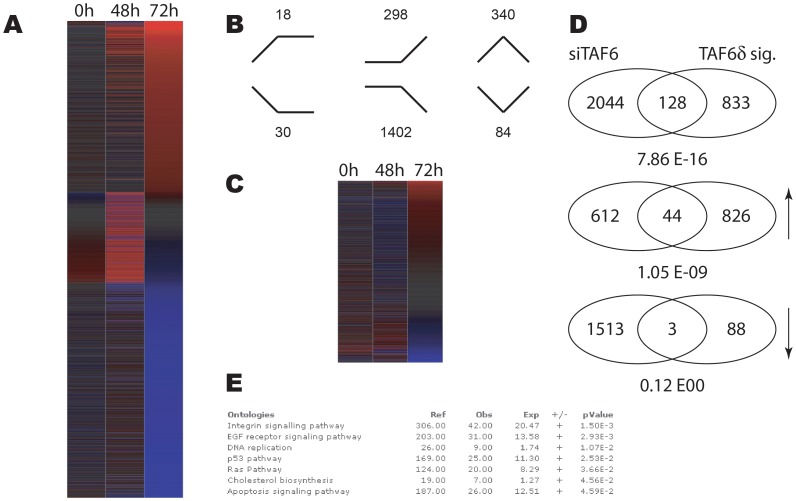
Distinct impact of TAF6δ induction versus total TAF6 mRNA depletion on the transcriptome of HeLa cells. (A) Heat map showing the impact of statistically significantly (p<0.05) changes in gene expression during TAF6 mRNA depletion by siRNA at 48 and 72 hours post transfection. Red indicates induction and blue repression. Genes were ordered according to fold change at 72 hours post transfection. (B) Distribution of expression profiles amongst the six possible outcomes. Genes upregulated or downregulated at the both time points are schematized with lines. (C) The previously defined TAF6δ transcriptome signature compared to the transcriptome resulting from depletion of total TAF6 mRNA. The heat map shows the gene expression during siRNA-mediated total TAF6 mRNA depletion for the 961 TAF6δ signature genes. (D) Venn diagrams depicting genes subsets statistically significantly regulated by total TAF6 mRNA depletion versus by TAF6δ induction. Upper diagram contains all regulated genes, middle diagram includes induces genes (upward arrow) and the lower Venn diagram includes repressed genes (downward arrow). (E) Gene ontology analysis of statistically significantly regulated genes during total TAF6 mRNA depletion. Enriched pathways are shown with their associated p-values.

The knock-down of total TAF6 resulted in statistically significant changes in gene expression levels including a global reduction in transcription accompanied by the increase in a minority of mRNA transcripts 48 hours after siRNA transfection and particularly after 72 hours (Fig. 2A). Since siRNA can potentially have off-target effects, we sought to validate the specificity of the siRNA used by comparing the expression of a panel of genes with an independent siRNA directed against TAF6. The genes assayed showed comparable changes in response to treatment with both the most efficient siRNA, si1 and the less efficient siRNA, si2 (Fig. S3). These data are compatible with the interpretation that the gene expression measured by microarray is due largely to specific interference with TAF6 expression. We also compared the changes in gene expression of the 961 previously identified TAF6δ-dependent transcripts [2] to changes in response to siRNA depletion of total TAF6 and found that the two conditions result in distinct transcriptome landscapes (Fig. 2C). To examine more closely the changes resulting from the induction of TAF6δ versus depletion of TAF6, we filtered the microarray data to compare the overlap between the two sets of regulated transcripts. Of 961 TAF6δ-dependent transcripts 128 were also significantly regulated by the siRNA against TAF6 at either 48 or 72 hours (Fig. 2D). Of the 128 regulated transcripts the majority (81) were oppositely regulated (induction versus repression). 44 transcripts were induced by both the loss of TAF6 and the induction of TAF6δ, and the overlap between these genes was statistically significant (P = 1,05×10^−09^, hypergeometric distribution). In contrast, 1513 genes were repressed in the absence of TAF6 and only three transcripts were repressed in both conditions (Fig. 2D) and there was no statistically significant overlap between repressed genes. The microarray results revealed a minor subset of common effects in the induction of TAF6δ versus depletion of total TAF6 transcripts. Importantly, the data also reveal that the induction of TAF6δ drives a distinct pro-apoptotic gene expression program and therefore underscore the necessity for TAF6δ isoform expression to trigger this specific pathway of apoptosis. To further explore the effects of TAF6 depletion gene ontology analysis was applied. Of the seven pathways that are statistically overrepresented during TAF6 depletion, only three including the integrin signaling pathway, the p53 pathway and apoptosis signaling pathways were also found in the TAF6δ transcriptome signature (Fig. 2E) [2]. To extent the comparison of gene expression patterns we compared the loss of total TAF6 at the earlier time point of 48 hours of treatment with siRNA, since these changes could potentially represent direct TAF6 target genes, with the gain of TAF6δ (Fig. S4). The data reinforce the distinct impact of TAF6δ induction compared to loss of TAF6 (Fig. S4). Taken altogether, the microarray data underscore the fact that the simple loss of total TAF6 mRNA does not recapitulate the same transcriptome impact that is observed when splicing of TAF6δ is selected at the expense of TAF6α expression.

### Development and validation of a minigene system to study TAF6 alternative splicing

**Figure 3:**
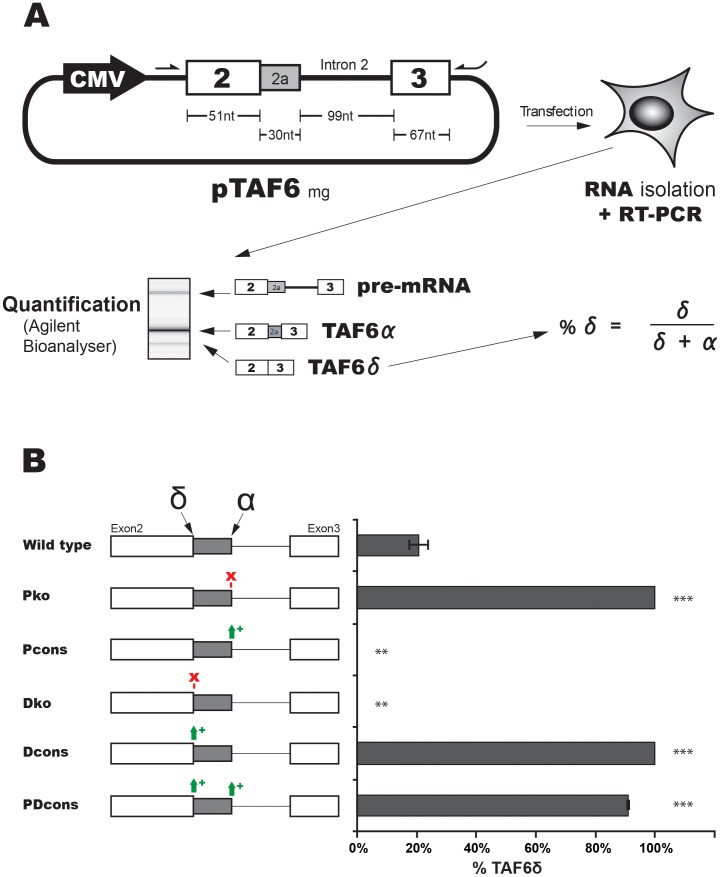
Development and validation of a minigene system to study the alternative splicing of TAF6. (A) A schematic diagram showing the workflow used to study *cis*-acting RNA sequences in TAF6 alternative splicing using a TAF6 minigene plasmid. The plasmid containing an uninterrupted genomic sequence from the *taf6* gene that includes portions of exons 2 and 3, as well as the natural intron 2 is depicted alone with the positions of primers used to detect exogenously expressed RNA species by RT-PCR. Minigenes were transfected into HeLa and 42 hours later total RNA was isolated for use in RT-PCR with primers from flanking plasmid sequences. PCR products were quantified by analysis using an Agilent Bioanalyzer. The percentage TAF6δ is expressed as a ratio of total spliced TAF6 mRNAs (δ + α). (B) Validation of the minigene system via mutagenesis. The proximal (P) 5′ splice site (SS) and distal (D) 5′ SS are illustrated. Mutations that knock-out (ko) SS or strengthen by creating consensus (cons) SS and their impact on the percentage of TAF6δ produced (x-axis), are indicated. (P<0.05 = *; P<0.01 = **; P<0.001 = ***).

**Figure 4:**
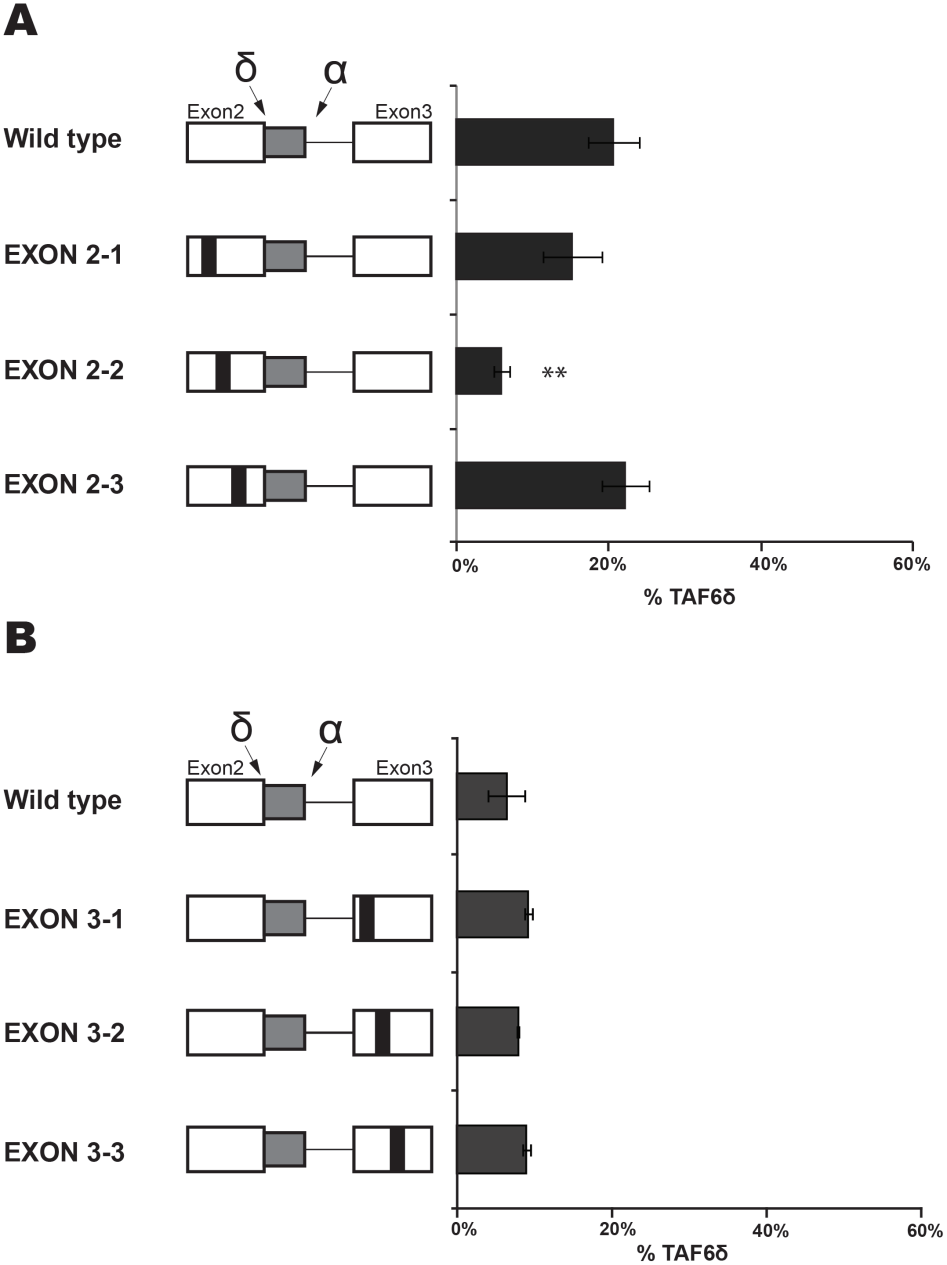
Scanning mutagenesis of constitutive exon 2 and exon 3. (A) Scanning mutations (black rectangles) in the TAF6 minigene were generated by PCR before transfection into HeLa. RNA was isolated and splice products were analysed by RT-PCR as in Figure 3. The percentage of exogenous TAF6δ mRNA produced by a given mutated construct are graphically shown (x-axis). (B) As in panel A, except that mutations were in exon 3. (P<0.05 = *; P<0.01 = **; P<0.001 = ***).

**Figure 5:**
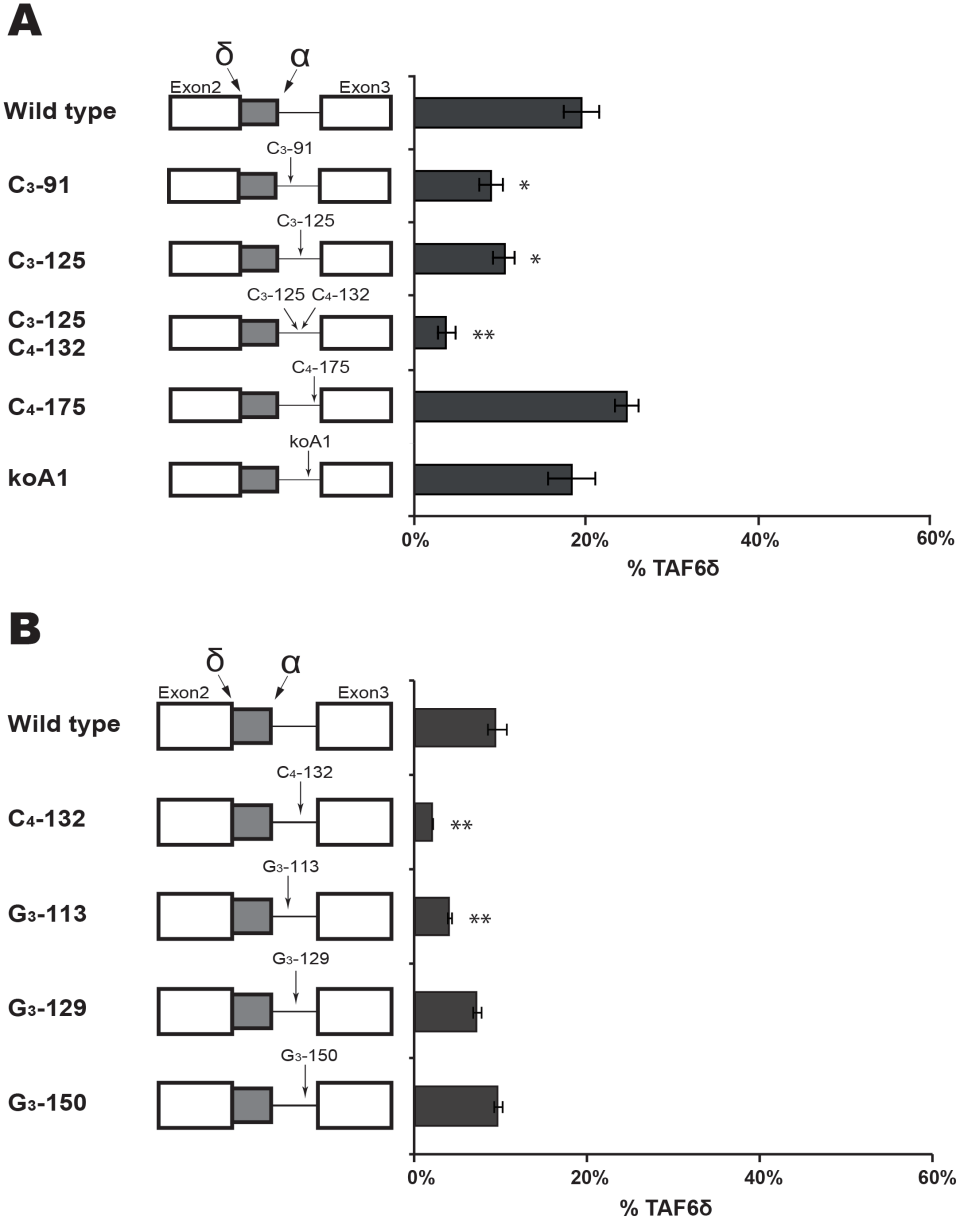
Targeted mutagenesis of intron 2 of the *taf6* gene. (A) Mutations of intron motifs are indicated with arrows in the TAF6 minigene constructs. HeLa cell transfection and splice product analysis was carried out as in Figure 3. The percentage of exogenous TAF6δ mRNA is graphically shown (x-axis). (B) As in panel A except mutations were focused on poly G motifs found within intron 2. (P<0.05 = *; P<0.01 = **; P<0.001 = ***).

**Figure 6:**
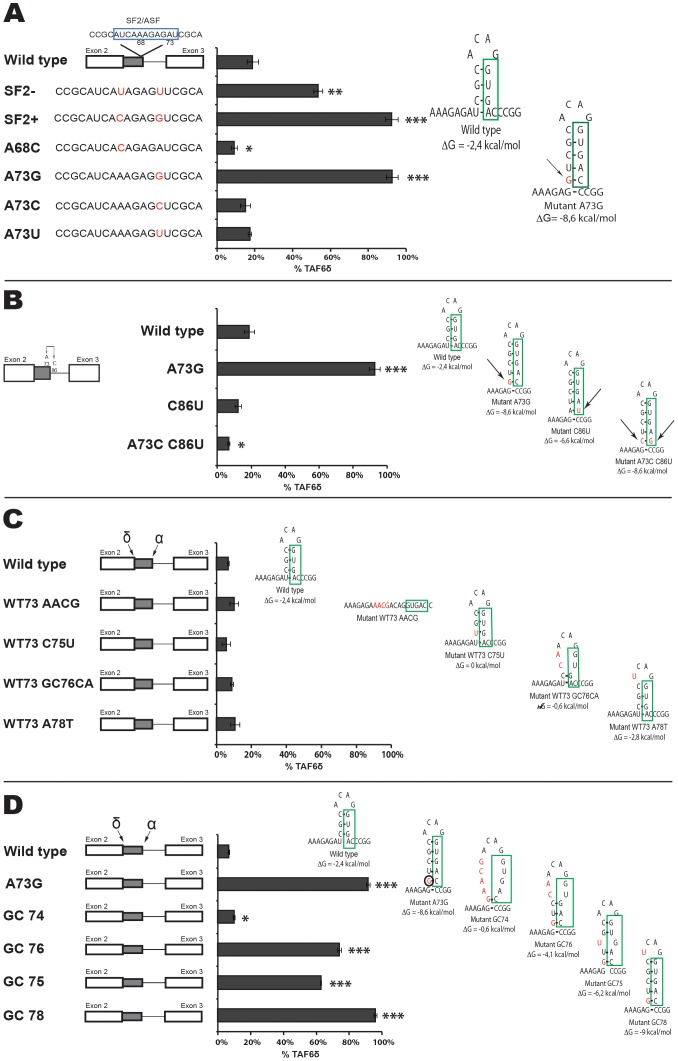
RNA secondary structure at the proximal 5′ splice site can force the selection of TAF6δ. (A) The sequence and names of mutations within alternative exon 2 (2a) of the TAF6 minigene constructs are indicated at the left. A potential SF2/ASF binding site is indicated by a blue box. HeLa cell transfection and splice product analysis was carried out as in Figure 3. The percentage of exogenous TAF6δ mRNA is graphically shown (x-axis). (B) As in panel A except that mutations (red nucleotides) are shown to the right in hypothetical RNA secondary structures generated using the M-Fold algorithm. The proximal 5′ splice site (SS) is indicated as green boxes. (C) As in B with further mutations. (D) As in B with further mutations. (P<0.05 = *; P<0.01 = **; P<0.001 = ***).

**Figure 7:**
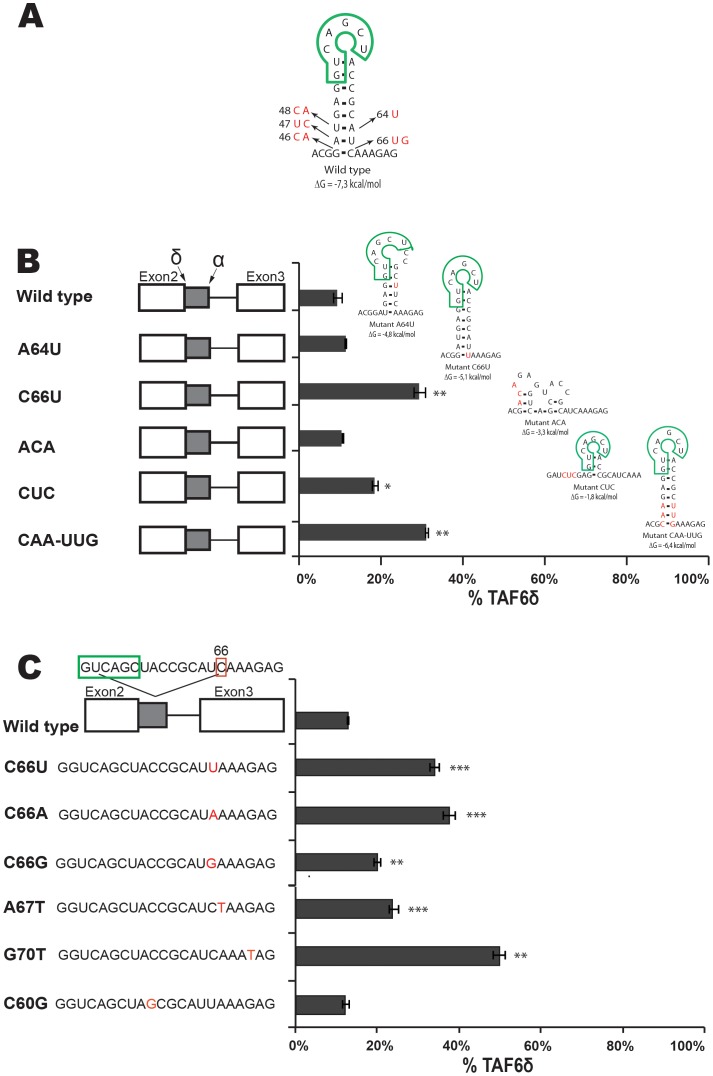
Evidence for an exonic splicing silencer in alternative exon 2. (A) A hypothetical RNA structure generated by M-fold is illustrated along with the position of the proximal TAF6 5′ splice site (green box). Selected mutation are indicated with arrows (red text) (B) The names of mutations within alternative exon 2 (2a) of the TAF6 minigene constructs are indicated at the right. HeLa cell transfection and splice product analysis was carried out as in Figure 3. The percentage of exogenous TAF6δ mRNA is graphically shown (x-axis). Mutations (red nucleotides) are shown to the right in hypothetical RNA secondary structures generated using the M-Fold algorithm. (C) As in panel A except that mutations (red nucleotides) are shown to the left. (P<0.05 = *; P<0.01 = **; P<0.001 = ***).

Having established the importance of the induction of TAF6δ versus the loss of TAF6α in driving a specific transcriptome profile and cell death programme, we next sought to investigate the molecular mechanisms that control the expression of the minor TAF6δ splice variant versus the major TAF6α splice variant. We cloned the genomic region from the TAF6 gene that contains part of exon 2, the alternative portion of exon 2, the natural full-length intron 2, and a part of exon 3 into the eukaryotic expression pcDNA3.1+ under the control of the CMV promoter (Fig. 3A). No deletions, truncations or other modifications to the short (99 nucelotides) intron were performed so that it retains the full-length endogenous sequence. HeLa cells were chosen as a model to study TAF6 alternative splicing since the pro-apoptotic TAF6δ splice variant was originally cloned from a HeLa cell cDNA library [1]. PCR primers specific to the transcribed flanking vector sequences were used to amplify exogenous TAF6 transcripts after transient transfection of the minigene into HeLa cells (Fig. 3A). PCR products corresponding to the expected sizes for the minigene products of unspliced pre-messenger, the major TAF6α transcript, and the minor TAF6δ transcript were detected by RT-PCR (Fig. 3A). Sequencing of each of these PCR products showed that they corresponded exactly to the expected splice products (B.B., unpublished results). Importantly, the ratio of TAF6δ versus total transcripts was found to range from 10% to 20% between independent transfections (Figs. 3B, 4A and 4B), a percentage that corresponds well to the endogenous TAF6 splicing pattern in HeLa cells [3]. The growth conditions of independent transfections appeared to influence basal levels of TAF6δ since biological triplicates within a single wave of transfections were highly reproducible (Figs. 3–7). Taken together, these data indicate that the plasmid minigene system accurately recapitulates endogenous TAF6δ splicing patterns in transfected HeLa cells.

To further characterize the minigene system we tested constructs bearing point mutations within one of the two alternative 5′ splice sites (SSs). When we crippled the proximal (α) 5′ SS by mutating the first two nucleotides of the intronic SS from the consensus GT to CA, the transfected minigene spliced uniquely at the alternative distal (δ) 5′ SS, as expected (Fig. 3B, Pko). Likewise, the same crippling mutation of the distal (δ) 5′ SS resulted in splicing in HeLa cells uniquely at the proximal (α) 5′ SS (Fig. 3B, Dko). We used a web tool http://rulai.cshl.edu/new_alt_exon_db2/HTML/score.html provided by Zhang laboratory (Cold Spring Harbor Laboratories) to calculate the alternative distal 5′ SS and the constitutive proximal 5′ SS strength scores as 2.8 and 6.4 respectively. These are relatively weak given that a perfect consensus site score is 12.6 and the average value for a constitutive 5′ SS is 8.1. To test the impact of splice site strength we mutated both sites individually to the consensus (AG/GUAAGU). When the proximal 5′ SS is changed to the consensus no residual detection of the alternative δ variant was detected (Fig. 3B, Pcons). When the alternative distal 5′ SS was changed to a consensus sequence all of the splice products used the distal site (Fig. 3B, Dcons). We conclude that one parameter that impacts the alternative splice site choice of TAF6 is the complementarity of the 5′ SSs to U1 snRNP as expected. We further conclude that the minigene system we developed provides a useful system to dissect the *cis*-acting RNA elements that control the expression of TAF6δ.

Having established a minigene system to study TAF6 alternative splicing we next set out to perform a mutational dissection of the RNA elements within the minigene that impact the expression of the pro-apoptotic TAF6δ isoform. We have applied a combination of resources to guide our mutational analysis including predictive algorithms for RNA binding proteins and *cis*-acting elements including RESCUE-ESE [33], ESE finder [34], and “Splicing Rainbow” [35]. We also considered evolutionary conservation of intronic sequences and employed scanning mutagenesis to identify *cis*-acting RNA splicing elements. For simplicity we have subdivided the minigene into several sub-regions and our mutational analysis of each region is presented below.

### Scanning mutagenesis of constitutive exon 2 and exon 3

To search for *cis*-acting RNA elements we initially performed scanning by mutating blocks of 10 nucleotides within the constitutive portion of exon 2 (Fig. 4A). The mutation of two nucleotide blocks Exon 2-1 and Exon 2-3 had no significant effect on the TAF6 splicing pattern (Fig. 4A). Another nucleotide block in exon 2, exon 2-2 produced reproducible reductions in the levels of TAF6δ (Fig. 4A). These results evoke that a possible *cis*-acting RNA sequence within the exon 2-2 could favour the selection of the distal (δ) 5′ SS. We note that in HeLa cells the endogenous basal levels (∼10%) of TAF6δ do not produce detectable TAF6δ protein by Western blotting or immunofluorescence with sensitive antibodies, suggesting the exon 2-2 mutation would not be expected to have profound biological impacts.

We next used the same scanning mutagenesis to query whether exon 3 contained RNA elements important for the selection of TAF6δ. We mutated three 10 nucleotide blocks near the 3′ SS of our TAF6 minigene (Fig. 4B). We found no significant differences in the ratio of TAF6δ for any of the three mutations exon 3-1, 3-2 or 3-3 (Fig. 4B). We conclude that the region of exon 3 proximal to intron 2 does not play a major role in the selection of the TAF6δ splice variant.

### Targeted mutagenesis of intron 2 of the *taf6* gene

To begin characterizing intron 2 of the minigene, we first examined sequence homology between the ninety-nine nucleotide long natural human intron 2 and forty-six vertebrate species (http://genome.ucsc.edu/). Minimal informative sequence conservation was found within intron 2, however a small conserved motif that fits the degenerate yUnAy consensus [36] for a human branch point site was identified at nucleotides 153–157 (Fig. S5). To determine if the adenine at position 156 corresponds to the branchpoint we mutated it to a guanosine. Upon transfection in HeLa cells the mutated minigene showed profoundly reduced splicing activity (Fig. S6). The residual splicing activity is low but detectable and could represent the inefficient use of alternative neighboring adenines. We conclude that adenine 156 is essential for splicing of the TAF6 minigene and most likely represents the major branchpoint site in intron 2.

To further explore *cis*-acting RNA elements within intron 2 that could impact the selection of the distal 5′ SS, we chose candidate motifs after manual and software-assisted (see above) analysis of potential regulatory sequence motifs. We decided to test the roles of several sequence motifs in the TAF6 minigene system including poly C motifs [37], poly G motifs [38] and a potential binding site for hnRNPA1 [39] because these motifs have been previously shown to influence alternative splicing. The mutation of a potential hnRNPA1 binding site [40] within intron had no significant effect on the TAF6 splicing pattern (Fig. 5A, koA1). Likewise, a mutation of the CCCC (C_4_) motif at position 175–178 had little effect on TAF6 alternative splicing (Fig. 5A, C_4_-175). The mutation of a C_3_ motif at positions 125–127 and C_4_ combined significantly reduced levels of the TAF6δ splice variant (Fig. 5A, C_3_-125; C_4_-132). To determine if one of these motifs was more important for TAF6δ expression we mutated them individually and found that mutation of C_3_-125 alone caused a small but measurable reduction in TAF6δ levels (Fig. 5A, C_3_-125). Mutation of the C_4_-132 motif alone caused a reduction in TAF6δ expression similar to that of the double mutation (Fig. 5B, C_4_-132), suggesting that the C_4_-132 motif plays the predominant role in TAF6δ splice site selection. To complete our investigation of poly C motifs, we mutated a C_3_ motif at position 91–93 in the minigene and found it produced modestly reduced splice selection of TAF6δ (Fig. 5A, C_3_-91).

We next turned our attention to the potential function of three poly G sequences within intron 2 of the TAF6 minigene. The mutation of a G_3_ motif at position 113–115 resulted in reduced expression of the TAF6δ splice variant (Fig. 5B, G_3_-113). The mutation of a G_3_ motif at position 129–131 had little impact on TAF6 splicing ratios (Fig. 5B, G_3_-129). The mutation of a G_3_ motif at position 150–152 generated wild type splicing ratios. (Fig. 5B, G_3_-150). Taken together, our analysis of intron 2 of the TAF6 minigene define a putative branchpoint adenosine at position 156 that is essential for splicing, and shows that specific poly C and poly G motifs in the intron can enhance the selection of the pro-apoptotic TAF6δ splice form.

### RNA secondary structure at the proximal 5′ splice site can force the selection of TAF6δ

We then focused on the alternative exon 2 (exon 2a) a critical region of the minigene because it lies physically between the α and δ alternative 5′ splice sites (Fig. 3A), and because modified antisense RNA oligonucleotides that anneal to it can shift the splicing from the major to the pro-apoptotic δ form in living cells [3]. The ESEfinder algorithm [34] was employed and detected a potential SF2 binding site in exon 2a (Fig. 6A). To test a potential role for the SF2 motif in the 5′ splice site choice we designed two point mutations to prevent (Fig. 6A, SF2-) or enhance (Fig. 6A, SF2+) SF2 binding. Upon transfection these mutations both gave strong increases in the levels of the δ splice form (Fig. 6A), a result not compatible with a role for SF2 binding to this motif. To further dissect the impact of the two-nucleotide mutation within SF2+ that caused a complete reversal of the splicing pattern, we mutated these positions individually. Mutation of position 68 alone had little effect on the splicing pattern (Fig. 6A, A68C). In contrast, mutation of adenosine 73 to guanosine alone resulted in a complete shift towards the usage of the distal δ 5′ SS (Fig. 6A, A73G). Having ruled out a role for SF2, we sought an alternative hypothesis for the strong impact of this mutation. Given the well-documented importance of RNA secondary structure on alternative splicing [15], [17], [18], [41], we employed the M-Fold algorithm [42] to test for potential impacts on RNA structure. When compared to the wild type sequence the A73G mutation was predicted to form a stem-loop that based on precedence [43] would be stable enough to potentially block U1 snRNP from binding to the proximal 5′ splice site (Fig. 6A, right). To test the hypothesis that mutation A73G reverses TAF6 splicing via the formation of secondary structure we performed further mutagenesis to provide support for the putative G73 - C86 base pair in cells. Mutation of C86U in the wild type context A73 did not change TAF6 splicing pattern (Fig. 6B). We also performed mutations that are predicted to form a stem-loop of equal stability to A73G replacing the natural C86 within position 5 of the proximal 5′ splice site with G to increase its strength. This construct showed less δ splicing that the wild type (Fig. 6B mutant A73C-C86U), compatible with a competition between splice site strength and RNA secondary structure in cells. To test whether the potential weak secondary structure formed by the wild type sequence could impact basal TAF6 splicing ratios we performed a series of mutations that would weaken such a potential structure, but found no strict correlation between the stability of such a structure and splice site selection (Fig. 6C). We conclude that any secondary structure forming with the wild type pre-mRNA under normal cellular conditions is not strong enough to impact selection of the distal 5′ SS. To further confirm the putative structure formed by the A73G mutation we performed mutations that disrupt the structure while leaving the proximal 5′ SS untouched and found no change in splicing (Fig. 6D, GC 74). Mutations that weaken but do not ablate the putative A73G-induced stem-loop showed intermediate levels of distal 5′ SS usage (Fig. 6D, GC 75 & GC 76). A point mutation in the loop of the predicted stem-loop had no effect, as expected (Fig. 6D, GC 78). Taken collectively, these findings indicate that a stem-loop structure competing for the proximal 5′ SS can strongly enhance the use of the distal δ 5′ SS.

### Evidence for an exonic splicing silencer in alternative exon 2

Given that local secondary structure occurs at the proximal 5′ SS, and that it is located only 30 nucleotides from the distal 5′ SS, we postulated that competition between RNA structure and the distal 5′ SS might also play a role in the expression of TAF6δ. We used the M-Fold algorithm [42] to predict potential secondary structures overlapping the distal 5′ SS. The most probable theoretical stem-loop structure predicted could potentially occlude the interaction of U1 snRNP interaction with the distal δ 5′ SS (Fig. 7A). To provide evidence to confirm or exclude the existence of such a stem-loop we constructed a series of minigenes with mutations that would weaken, destroy or maintain it. No correlation was observed between potential stem-loop strength and the splicing outcome, ruling out a major role for such a structure in the regulation of TAF6 alternative splicing (Fig. 7A).

**Figure 8:**
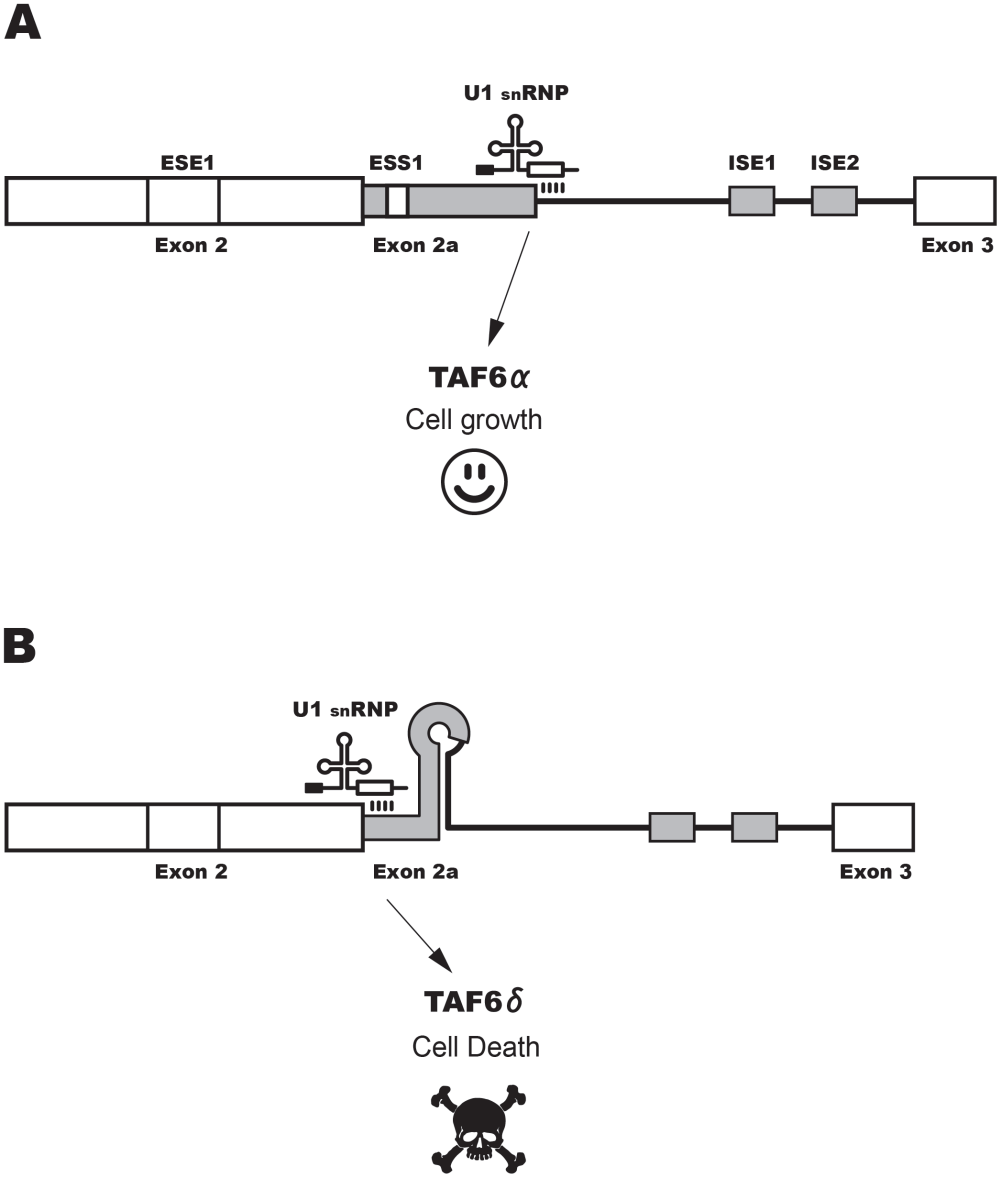
A hypothetical model for TAF6δ alternative splicing. (A) The TAF6 minigene construct is schematically shown with putative exonic splicing enhancer (ESE), exonic splicing silencer (ESS) and intronic splicing enhancer (ISE) motifs indicated with boxes. Enhancer or silencer definitions are given with respect to the pro-apoptotic TAF6δ isoform but likely act simultaneously to repress one 5′ SS while enhancing the other because of the small distance (30 nucleotides) between them. (B) As in panel A, except that mutations were focused on poly G motifs found within intron 2.

Interestingly, however, our mutational analysis revealed that a single nucleotide change (C66U) produced a greater than two-fold increase in the production of the TAF6δ splice form (Fig. 7A and B, C66U). To further delineate the role of cytosine 66 we mutated it to uridine, adenosine and guanosine respectively. Mutations C66U and C66A both resulted in clear increases in TAF6δ splice form production (Fig. 7C), even though these nucleotides have distinct hydrogen bonding specificities within RNA secondary structures. C66G had much less effect than these mutations, although the mutation would also be expected to have altered base-pairing within RNA structure. To further dissect a potential regulatory RNA sequence in the region of cytosine 66 we performed further point mutations. Changing adenosine 67 to thymine significantly increased TAF6δ splice selection (Fig. 7C, A67T). A nearby change, G70T, also increased TAF6δ splice selection (Fig. 7C). In contrast, a slightly more distant mutation C60G did not significantly change the splicing ratio (Fig. 7C). Taken together, the above results suggest that an exonic splicing silencer (ESS) is located in the region of nucleotides 66–70 within the alternative exon of TAF6. We found no evidence that RNA secondary structure within this region plays an important role in splice site choice. The mutational data described above support a hypothetical model for *cis*-acting RNA elements in the regulation of TAF6 alternative splicing that is presented schematically in Figure 8.

## Discussion

TAF6 is a core subunit of the general RNA Pol II transcription factor TFIID [5], and has previously demonstrated to be essential for viability in several organisms including the budding yeast *Saccharomyces cerevisiae* [28], [44], *Drosophila* fruit flies [30], the flowering plant *Arabidopsis thaliana* as well as the fresh water fish *Danio rerio* [31]. Here we show for the first time that TAF6 is essential for viability in human cells (Fig. 1). Although this result is not unexpected, the significance of the finding derives from the potential of TAF6 as a therapeutic target for numerous diseases that result from deregulated apoptosis [45]. Many apoptotic genes are required for normal development at the organismal level but tumor suppressors such as BRCA1, PTEN, CDKN2A (ARF/p16INK4), RB1, APC, and p53, Bcl-2 family members, the caspases and death receptors are all dispensable for viability at the cellular level [46], [47]. TAF6 therefore represents a rare class of genes that are essential for cell viability, but also possess splice variants with potent pro-apoptotic activity. As an essential gene with pro-apoptotic potential TAF6 is of high strategic interest in the development of anti-cancer treatments that avoid the development of chemoresistance and to target p53 negative tumors [48].

Using a minigene system that recapitulates the endogenous TAF6 splicing pattern we identified several *cis*-RNA elements that modulate TAF6δ splicing as schematically shown in Figure 8. The sensitivity of the proximal 5′ splice site evokes the possibility that the modulation of RNA folding could contribute to the physiological selection of TAF6δ. Further work will be required to confirm or exclude this hypothesis. The first mapping of the *cis*-acting RNA within elements provides the essential groundwork for future studies to identify *trans*-acting factors that regulate TAF6δ splicing. Indeed, the identification of the important RNA elements will be crucial for both proteomic [49] and genomic [50] approaches to identify *trans*-acting splice regulatory proteins. A limitation of the current study is the fact that the mutations could conceivably differentially alter RNA stability in addition to splice site choice. In addition potential long-range *cis*-acting RNA elements will not have been identified due to the limited size of our TAF6 minigene. Further work will be required to address these possibilities.

Accumulating evidence points to a potential link between the TAF6δ pathway and cancer biology. A cDNA encoding the major TAF6α variant was identified in a large-scale screen as being able to increase colony formation in human hepatocellular carcinoma cells and mouse embryonic fibroblasts [51]. Integrative genomic data show that the taf6 gene is amplified in lung cancer [52]. TAF6 has been reported as a genomic marker of poor prognosis in lung adenocarcinoma [53]. TAF6 mRNA was identified as being overexpressed in inflammatory breast cancer [54]. Interestingly, a specific splice variant of TAF6 with an extended exon 2 is reportedly overrepresented in ductal cell carcinoma [55]. Taken together these findings suggest that the major anti-apoptotic TAF6α splice variant possesses oncogenic potential. In stark contrast, the minor TAF6δ splice variant has pro-apoptotic activity evoking a potential tumor suppressor activity [1], [2], [3]. It is conceivable that anti-apoptotic TAF6α expression can be decoupled from pro-apoptotic TAF6δ in certain tumor types. The mapping of key *cis*-acting RNA elements we present here paves the way to experimentally test the existence of mutations in the essential taf6 gene that could reduce or prevent TAF6δ expression in human tumors.

## Supporting Information

Figure S1 The role of alternative splicing in the TAF6δ pathway of apoptosis. A schematic model depicts the exon 2, intron 2, exon 3 region of the *taf6* gene. Use of proximal 5′ splice site (SS) generates the major TAF6α isoform that dimerizes with its normal partner TAF9 within the TFIID complex resulting in a gene expression program allowing cell growth. Selection of the distal alternative 5′ SS removes 10 amino acids to generate TAF6δ that cannot interact with TAF9 but is incorporated into a TFIIDπ complex that drives a pro-apoptotic gene expression and consequently cell death.
(TIF)

Figure S2 Endogenous TAF6δ is not detectable in HeLa cells under normal growth conditions. (A) Protein samples from HeLa cells that were transfected with a scrambled (Ctl), TAF6-1 (si1) or TAF6-2 (si2) siRNA were used to perform western blots. The resulting membranes were incubated with either a TAF6α or a TAF6 total targetting antibody. (B) The TAF6α and TAF6δ specific antibodies were used to detect the protein in lysates of untransfected HeLa cells (NT). Protein extracts of mock (M), empty vector (EV), TAF6δ (δ) of TAF6α (α) transfected cells were used as controls. (C) Overexposure of membranes incubated with two different TAF6δ antibodies show no signal in the untransfected cells. The 37TA 2D5 antibody, which was raised against the δ isoform, but also recognizes TAF6α, detects no protein in non-transfected cells. The 37TA 1C2 antibody is highly specific for TAF6δ. The white asterisk indicates an non-specific band that migrates slightly slower than the δ splice variant.
(TIF)

Figure S3 Validation of TAF6 siRNA specificity. Quantitative real-time PCR was used to assess the similarity of gene regulation 48 h after the transfection by two different siRNAs targetting TAF6.
(TIF)

Figure S4 Distinct impact of TAF6δ induction versus total early (48 hour) TAF6 mRNA depletion on the transcriptome of HeLa cells. (A) Heat map comparing the impact of statistically significantly (p<0.05) changes in gene expression during TAF6 mRNA depletion by siRNA at 48 hours post transfection to the TAF6delta expression profile. Red indicates induction and blue repression. Genes were ordered independently according to fold change. (B) Gene ontology analysis of statistically significantly regulated genes during total TAF6 mRNA depletion at 48 hours post-transfection. Enriched pathways are shown with their associated p-values. (C) Venn diagram depicting genes statistically significantly regulated by total TAF6 mRNA depletion versus TAF6δ induction. (D) Logarithmic fold-changes of genes regulated statistically significantly by TAF6 mRNA depletion 48 hours post-transfection and by TAF6δ induction are shown side by side.
(TIF)

Figure S5 Schematic representation of mutations used in this study. (A) The TAF6 minigene construct is shown schematically. (B) A nucleotide resolution list of mutations (altered nucleotides shown in red) from different regions of the minigene are illustrated. Black text corresponds to wild type sequences and the exons 2 and 3 (white boxes), alternative exon 2a (grey box) and intron 2 (black line) are indicated above the sequences.
(TIF)

Figure S6 Mapping of the branchpoint in intron 2 of the TAF6 minigene. (A) A TAF6 minigene construct bearing a point mutation in a putative branchpoint (adenine 156 to guanosine) was transfected into HeLa cells for splicing analysis as in Figure 3. The pre-mRNA as well as the spliced products are indicated with arrows. (B) Exogenously expressed TAF6 pre-mRNA and spliced products were quantified as in Figure 3 and the percentage of exogenous TAF6 minigene splicing is graphically shown (y-axis).
(TIF)

Table S1 List of synthetic oligonucleotides used in this study. The names of mutations are given in the left column and the oligonucleotide sequence is listed in the right column.
(XLSX)
