# Supplementary figures and images for: Alternative Splicing of TAF6: Downstream Transcriptome Impacts and Upstream RNA Splice Control Elements
Source: PLoS One. 2014 Jul 15;9(7):e102399. doi: 10.1371/journal.pone.0102399 (PMC4099370; doi:10.1371/journal.pone.0102399)

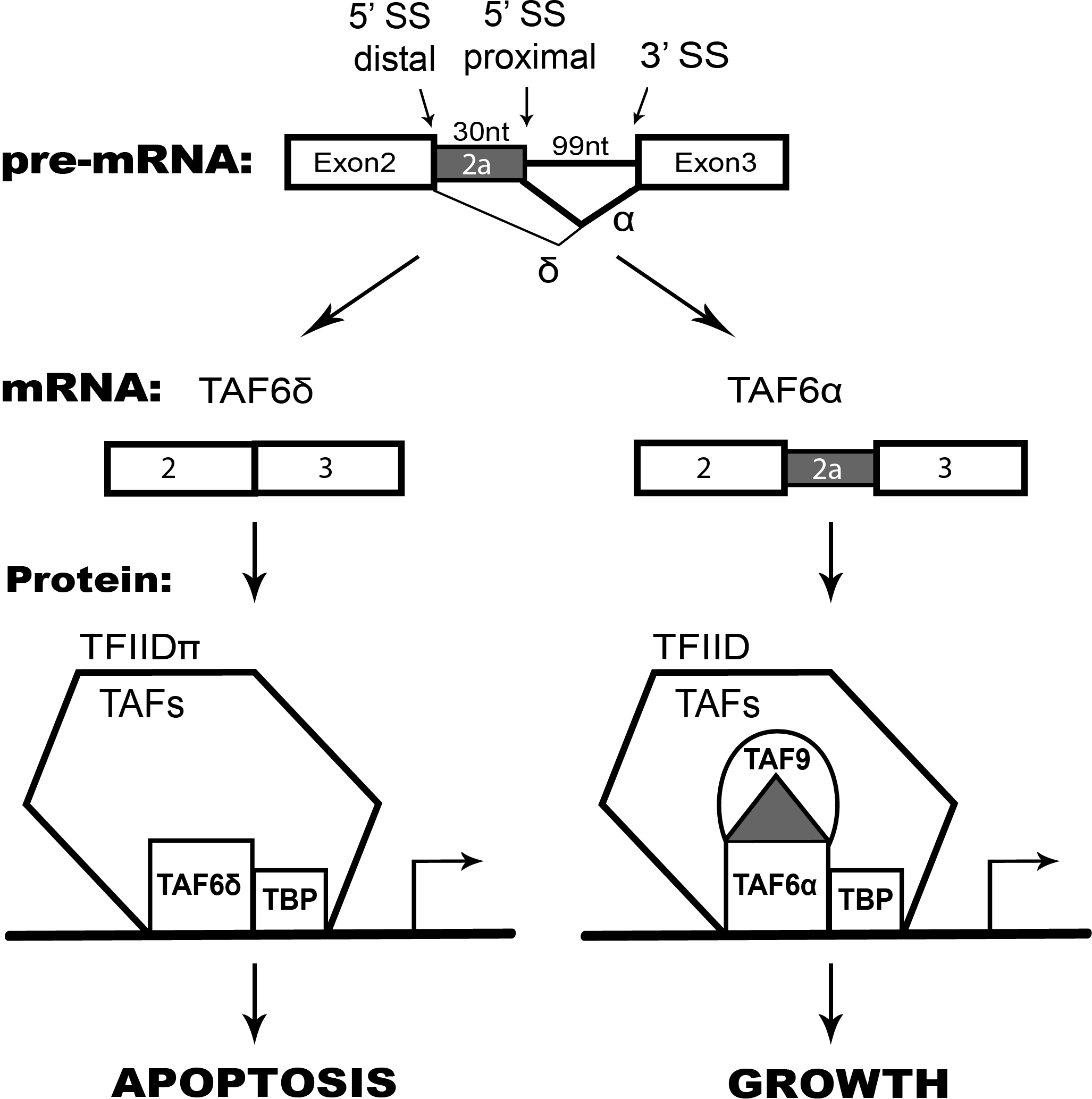

Supplement: Figure S1 — The role of alternative splicing in the TAF6δ pathway of apoptosis. A schematic model depicts the exon 2, intron 2, exon 3 region of the taf6 gene. Use of proximal 5′ splice site (SS) generates the major TAF6α isoform that dimerizes with its normal partner TAF9 within the TFIID complex resulting in a gene expression program allowing cell growth. Selection of the distal alternative 5′ SS removes 10 amino acids to generate TAF6δ that cannot interact with TAF9 but is incorporated into a TFIIDπ complex that drives a pro-apoptotic gene expression and consequently cell death. (TIF) [file pone.0102399.s001.tif]

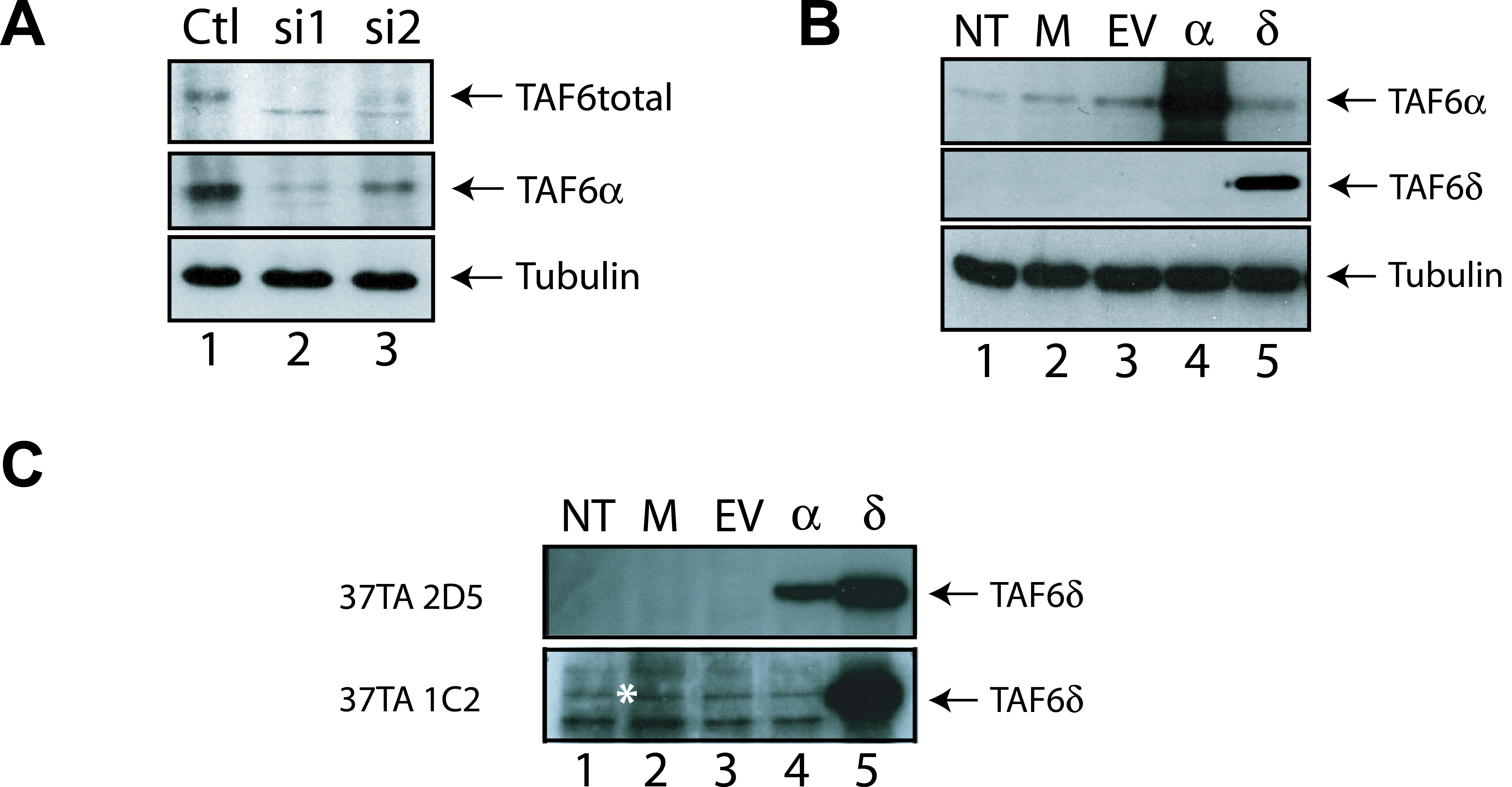

Supplement: Figure S2 — Endogenous TAF6δ is not detectable in HeLa cells under normal growth conditions. (A) Protein samples from HeLa cells that were transfected with a scrambled (Ctl), TAF6-1 (si1) or TAF6-2 (si2) siRNA were used to perform western blots. The resulting membranes were incubated with either a TAF6α or a TAF6 total targetting antibody. (B) The TAF6α and TAF6δ specific antibodies were used to detect the protein in lysates of untransfected HeLa cells (NT). Protein extracts of mock (M), empty vector (EV), TAF6δ (δ) of TAF6α (α) transfected cells were used as controls. (C) Overexposure of membranes incubated with two different TAF6δ antibodies show no signal in the untransfected cells. The 37TA 2D5 antibody, which was raised against the δ isoform, but also recognizes TAF6α, detects no protein in non-transfected cells. The 37TA 1C2 antibody is highly specific for TAF6δ. The white asterisk indicates an non-specific band that migrates slightly slower than the δ splice variant. (TIF) [file pone.0102399.s002.tif]

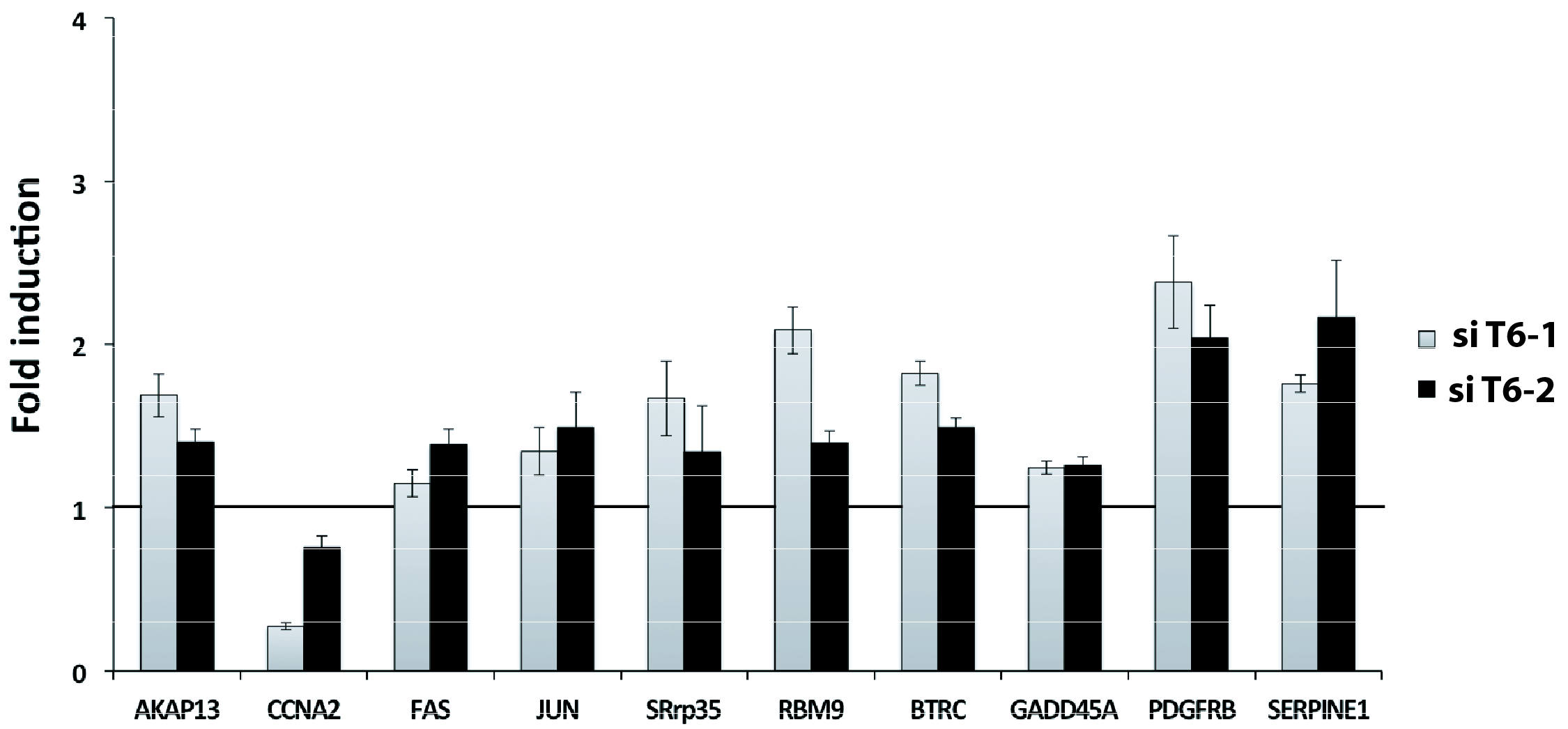

Supplement: Figure S3 — Validation of TAF6 siRNA specificity. Quantitative real-time PCR was used to assess the similarity of gene regulation 48 h after the transfection by two different siRNAs targetting TAF6. (TIF) [file pone.0102399.s003.tif]

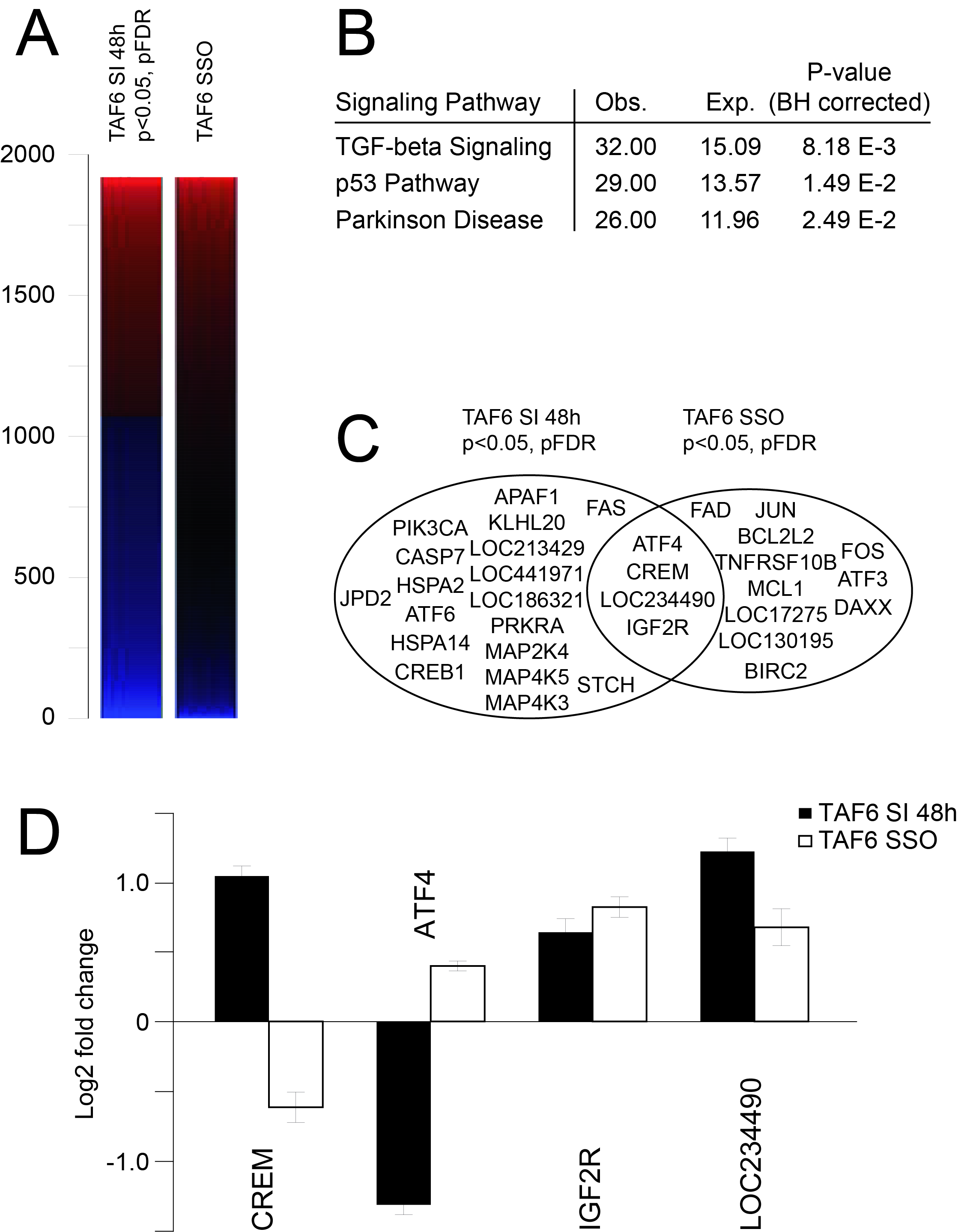

Supplement: Figure S4 — Distinct impact of TAF6δ induction versus total early (48 hour) TAF6 mRNA depletion on the transcriptome of HeLa cells. (A) Heat map comparing the impact of statistically significantly (p<0.05) changes in gene expression during TAF6 mRNA depletion by siRNA at 48 hours post transfection to the TAF6delta expression profile. Red indicates induction and blue repression. Genes were ordered independently according to fold change. (B) Gene ontology analysis of statistically significantly regulated genes during total TAF6 mRNA depletion at 48 hours post-transfection. Enriched pathways are shown with their associated p-values. (C) Venn diagram depicting genes statistically significantly regulated by total TAF6 mRNA depletion versus TAF6δ induction. (D) Logarithmic fold-changes of genes regulated statistically significantly by TAF6 mRNA depletion 48 hours post-transfection and by TAF6δ induction are shown side by side. (TIF) [file pone.0102399.s004.tif]

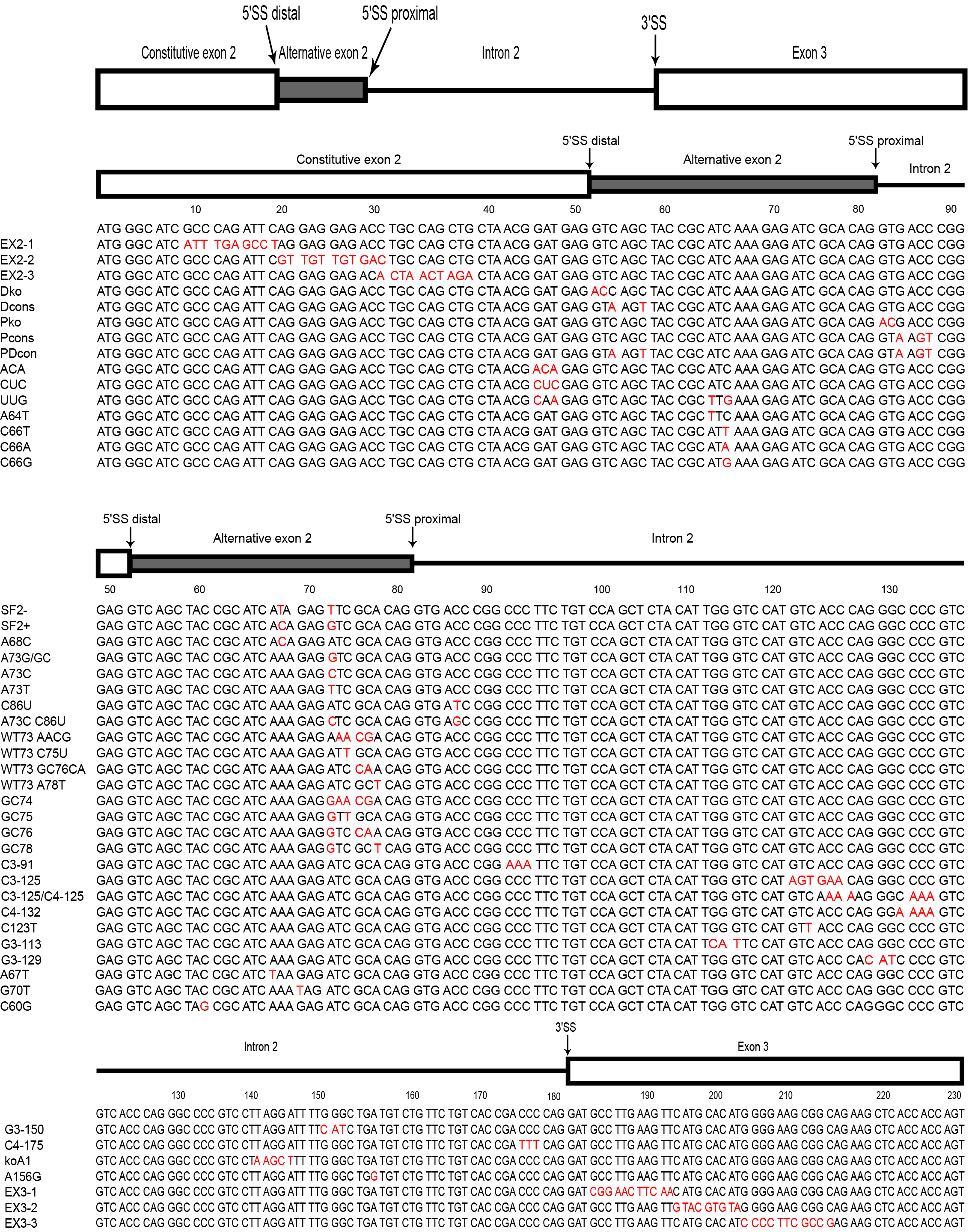

Supplement: Figure S5 — Schematic representation of mutations used in this study. (A) The TAF6 minigene construct is shown schematically. (B) A nucleotide resolution list of mutations (altered nucleotides shown in red) from different regions of the minigene are illustrated. Black text corresponds to wild type sequences and the exons 2 and 3 (white boxes), alternative exon 2a (grey box) and intron 2 (black line) are indicated above the sequences. (TIF) [file pone.0102399.s005.tif]

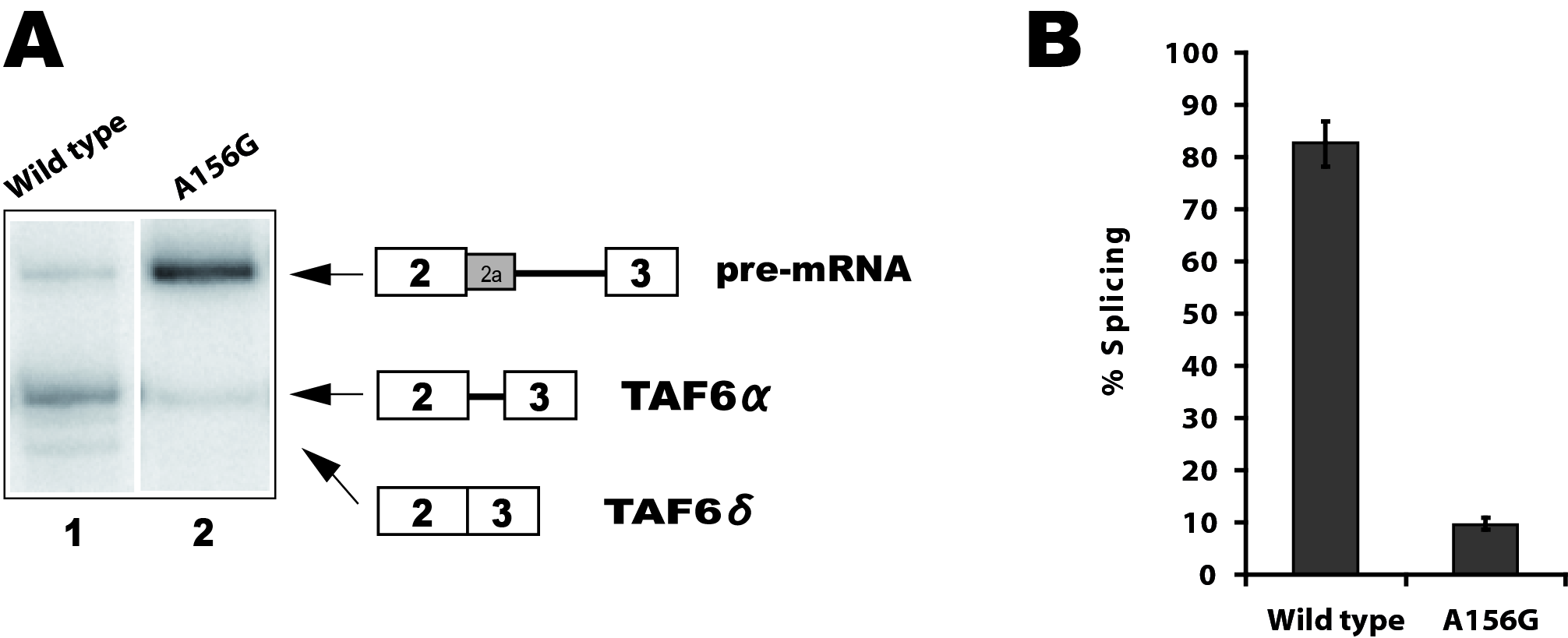

Supplement: Figure S6 — Mapping of the branchpoint in intron 2 of the TAF6 minigene. (A) A TAF6 minigene construct bearing a point mutation in a putative branchpoint (adenine 156 to guanosine) was transfected into HeLa cells for splicing analysis as in Figure 3. The pre-mRNA as well as the spliced products are indicated with arrows. (B) Exogenously expressed TAF6 pre-mRNA and spliced products were quantified as in Figure 3 and the percentage of exogenous TAF6 minigene splicing is graphically shown (y-axis). (TIF) [file pone.0102399.s006.tif]
